# Intravitreal aflibercept and ranibizumab for pachychoroid neovasculopathy

**DOI:** 10.1038/s41598-019-38504-y

**Published:** 2019-02-14

**Authors:** Byung Ju Jung, Joo Young Kim, Jae Hyung Lee, Jiwon Baek, Kook Lee, Won Ki Lee

**Affiliations:** 1 Department of Ophthalmology and Visual Science, Seoul St. Mary’s Hospital, College of Medicine, The Catholic University of Korea, 505 Banpo-dong, Seocho-ku, Seoul, 137-701 Korea; 2 Department of Ophthalmology and Visual Science, Bucheon St. Mary’s Hospital, College of Medicine, The Catholic University of Korea, Seoul, Korea

## Abstract

This retrospective study was to compare the efficacy of intravitreal injection of ranibizumab and aflibercept for patients with pachychoroid neovasculopathy. 54 eyes were initially treated with 3 monthly loading injections of ranibizumab or aflibercept. Treatment switching from ranibizumab to aflibercept, and aflibercept to photodynamic therapy was done at 3 months in case of incomplete fluid absorption. At 3 months, the rate of complete fluid absorption was significantly higher in the aflibercept-treated group than in the ranibizumab-treated group (82.6% vs 51.6%, *p* = 0.018). The mean reduction of subfoveal choroidal thickness was significantly greater in the aflibercept group than in the ranibizumab group (−35 µm vs −9 µm, *p* = 0.013). There was no significant difference between the two groups in terms of visual improvement or decrease in central macular thickness. Complete fluid absorption was achieved after switching from ranibizumab to aflibercept in 13 of 15 eyes (86.7%). Adjunctive photodynamic therapy was required in 6 eyes. In conclusion, treatment mainly with anti-vascular endothelial growth factor effectively improved visual acuity within 12 months (from 20/56 to 20/44 at 3 months and to 20/36 at 12 months). Aflibercept was superior to ranibizumab in achieving dry macula and reducing choroidal thickness at 3 months.

## Introduction

The term “pachychoroid neovasculopathy” has been introduced to describe Type 1 neovascularization associated with choroidal thickening and/or dilated Haller vessels in the absence of characteristic age-related macular degeneration features^1,2^. It is a newly recognized clinical entity of choroidal neovascularization (CNV) that is thought to be induced by pachychoroid phenotype–driven pathophysiological mechanisms^3^. This disease entity has been suggested to be in the spectrum between central serous chorioretinopathy (CSC) and polypoidal choroidal vasculopathy (PCV). Pang and Freund reported that pachychoroid neovasculopathy can develop after long-standing CSC and may eventually progress to PCV in some cases^2^.

However, pachychoroid neovasculopathy still remains a subjective term with no clear definition. Moreover, in some reports, type 1 neovascularization with polypoidal lesions was included in the inclusion criteria of pachychoroid neovasculopathy, causing confusion in the classification of diseases^4–6^. The polypoidal lesion is a hallmark of PCV, and based on the diagnostic criteria to date, Type 1 neovascularization that accompanies polypoidal lesions shown by indocyanine green angiography (ICGA) should be classified as PCV and not otherwise^7^. Based on the original description, pachychoroid neovasculopathy should be designated in neovascular lesions with pachychoroid features but without polypoidal lesions. In the current study, we tried to apply finer and more detailed criteria in defining pachychoroid neovasculopathy, although no criteria have been established for this new disease entity.

The emergence of anti-vascular endothelial growth factor (VEGF) agents has led to a significant reduction in visual impairment from neovascular age-related macular degeneration (nAMD)^8^, and ranibizumab and aflibercept are the two approved drugs. Results from randomized clinical trials indicated that monthly doses of ranibizumab produced similar efficacy and safety outcomes as aflibercept (dosed either monthly or every 2 months, following 3 initial monthly loading doses) in nAMD^9^. However, ranibizumab and aflibercept differ with respect to their molecular structure, and may produce different efficacy in specific diseases^10^. The response of pachychoroid neovasculopathy to anti–VEGF drugs has not been fully understood yet. A few studies recently reported efficacy of anti-VEGF therapy in pachychoroid neovasculopathy similar to that in nAMD^6,11^. However, enrolled cases were relatively small and PCV lesions were included in both groups, making their results inconclusive. Moreover, there have been no reports specifically focusing on the differences between the two anti-VEGF agents in the treatment of pachychoroid neovasculopathy.

The purpose of this study was to evaluate 1-year vision improvement and morphological changes with the use of mainly anti-VEGF drugs for pachychoroid neovasculopathy. Also we tried to determine the differences between aflibercept and ranibizumab in terms of short-term therapeutic efficacy in the treatment of this disease.

## Results

### Baseline characteristics

A total of 54 eyes (52 patients) were diagnosed with pachychoroid neovasculopathy according to our inclusion criteria. The mean age of the entire study group was 64.0 ± 7.6 years. The mean subfoveal choroidal thickness (SFCT) was 436 ± 83 µm (range: 307–599 µm) at baseline, and the choroidal vascularity index (CVI) was 65.0 + 2.7%. On ICGA, 34 eyes (64.1%) demonstrated choroidal vascular hyperpermeability (CVH), and 7 eyes (12.7%) had retinal pigment epithelium (RPE) atrophic tracks on fluorescein angiography (FAG) or fundus autofluorescence (FAF). The intraclass correlation coefficients for inter-observer reproducibility about CVI showed almost perfect agreement with the level of 0.93 (95%CI: 0.87–0.96, P < 0.001). The Fleiss’ kappa test also demonstrated substantial agreement with the level of 0.77 (P < 0.001) in the analysis of CVH. Fellow eye findings of 50 patients were pachychoroid pigment epitheliopathy in 17 eyes, chronic CSC without choroidal neovascularization in 2 eyes, and PCV in 1 eye. The baseline characteristics of the study eyes are listed in Table 1. At baseline, there were no significant differences between the aflibercept-group (23 eyes) and the ranibizumab-group (31 eyes) with regard to best-corrected visual acuity (BCVA), central macular thickness (CMT), SFCT, greatest linear dimension, CVI, and incidence of CVH.

**Table 1:** Baseline characteristics of the patients with pachychoroid neovasculopathy enrolled in this study.

	Total (n = 55)	Ranibizumab (n = 31)	Aflibercept (n = 23)	*P* value
Age, years	64.0 ± 7.6	64.6 ± 8.4	63.1 ± 6.2	0.672^a^
Gender, male/female	35/20	19/12	16/7	0.859^b^
RPE tract, n (%)	7 (12.7)	4 (12.9)	3 (13.0)	0.844^b^
Greatest linear diameter of CNV, µm	2782 ± 775	2837 ± 850	2697 ± 660	0.747^a^
Choroidal vascular hyperpermeability, n (%)	34 (64.1)	18 (58.1)	16 (69.5)	0.543^b^
Central macular thickness, µm	337 ± 53	332 ± 54	345 ± 52	0.456^a^
Subfoveal choroidal thickness, µm	436 ± 83	438 ± 78	433 ± 93	0.877^a^
Choroidal vascularity index, %	65.0 + 2.7	65.1 ± 2.5	64.9 ± 3.1	0.687^a^
BCVA, logMAR	0.45 ± 0.26	0.46 ± 0.24	0.43 ± 0.26	0.614^a^

RPE = retinal pigment epithelium; CNV = choroidal neovascularization; BCVA = best-corrected visual acuity; logMAR = logarithm of the minimal angle of resolution.

^a^Mann-Whitney test.

^b^Chi-square test.

### Anatomical and functional outcomes at 3 months

Table 2 summarizes the anatomical and visual outcomes of the two groups. The proportion of patients with complete fluid absorption was significantly higher in the aflibercept group (19 of 23 eyes, 82.6%) than in the ranibizumab group (16 of 31 eyes, 51.6%, P = 0.018).

**Table 2:** Clinical outcomes after ranibizumab and aflibercept injections at 3 months.

	Ranibizumab (n = 31)	Aflibercept (n = 23)	*P* value
Complete resolution of fluid, n (%)	16(51.6)	19(82.6)	0.018^b^
BCVA, logMAR			
baseline	0.46 ± 0.24	0.43 ± 0.26	0.614^a^
3 months	0.38 ± 0.31(0.002)^c^	0.27 ± 0.21(0.013)^c^	0.287^a^
Central macular thickness, μm			
baseline	332 ± 54	345 ± 52	0.456^a^
3 months	276 ± 60(<0.001)^c^	264 ± 83(0.004)^c^	0.258^a^
Subfoveal choroidal thickness, μm			
baseline	438 ± 78	433 ± 93	0.877^a^
3 months	429 ± 75(0.042)^c^	399 ± 96(<0.001)^c^	0.371^a^

BCVA = best-corrected visual acuity; logMAR = logarithm of the minimal angle of resolution.

^a^Mann-Whitney test.

^b^Chi-square test.

^c^Wilcoxon signed rank test.

The mean SFCT decreased significantly from 433 ± 93 µm to 399 ± 96 µm in the aflibercept group, and from 438 ± 78 µm to 429 ± 75 µm in the ranibizumab group (P < 0.001, P = 0.042, respectively). The mean reduction was significantly greater in the aflibercept group than in the ranibizumab group (−35 ± 34 µm vs. −9 ± 22 µm, P = 0.013). The mean CMT decreased significantly from 345 ± 52 µm to 264 ± 83 µm in the aflibercept group, and from 332 ± 54 µm to 276 ± 60 µm in the ranibizumab group (P = 0.004, P < 0.001, respectively). The mean CMT at 3 months did not differ significantly between the two groups (P = 0.258).

Significant visual improvement was seen in both groups. The mean the logarithm of the minimal angle of resolution (logMAR) BCVA was 0.43 ± 0.26 logMAR (Snellen equivalent, 20/54) at baseline and 0.27 ± 0.21 logMAR (Snellen equivalent, 20/37) at 3 months in the aflibercept group (P = 0.013) and 0.46 ± 0.24 logMAR (Snellen equivalent, 20/58) at baseline and 0.38 ± 0.31 logMAR (Snellen equivalent, 20/50) at 3 months in the ranibizumab group (P = 0.002). At 3 months, no statistically significant difference was noted between the two groups regarding logMAR BCVA (P = 0.287) or CMT (P = 0.258).

### Treatment switch and clinical outcomes at 12 months

In 35 eyes that showed complete fluid absorption at 3 months (19 eyes in the aflibercept group, 16 eyes in the ranibizumab group), 7 eyes (36.8%) in the aflibercept group and 9 eyes (56.2%) in the ranibizumab group showed recurrent fluid accumulation on spectral-domain optical coherence tomography (SD OCT) within 12 months (P = 0.163). The favorable response to initial anti-VEGF drugs was maintained in these eyes, and treatment switch was not required until 12 months. The mean number of injections was 3.5 (range: 3 to 6, median: 3) in the aflibercept group and 4.6 (range: 3 to 8, median: 3) in the ranibizumab group until 12 months (P = 0.114).

**Figure 1:**
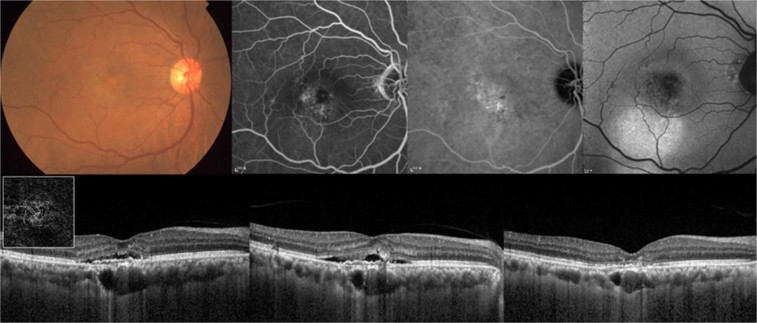
The case of a 69-year-old male who was switched to aflibercept injections due to the poor response to ranibizumab. (Top left) Color fundus photograph shows reduced background fundus tessellation without drusen. (Top second) Fluorescein angiography demonstrates ill-defined late leakage. (Top third) Late-phase indocyanine green angiography exhibits focal hyperfluorescent spots and choroidal vascular hyperpermeability within the posterior pole. (Top right) Fundus autofluorescence image shows hyperfluorescence in the area inferior to the fovea. (Bottom left) Spectral-domain optical coherence tomography (OCT) at baseline reveals Type 1 neovascularization, which was confirmed on swept-source OCT angiography (inlet). Multiple dilated Haller vessels with attenuation of choriocapillaris are seen beneath the lesion. Best-corrected visual acuity (BCVA) of the right eye was 20/63 at baseline. (Bottom middle) After three loading injections of ranibizumab, subretinal fluid remained and BCVA decreased to 20/80. (Bottom right) Treatment was switched to aflibercept, and after three consecutive injections, complete fluid absorption was achieved and BCVA improved to 20/40. The subfoveal choroidal thickness decreased from 354 µm at baseline, to 341 µm after ranibizumab injections, and 293 µm after aflibercept injections.

In 13 of 15 eyes (86.7%) that did not respond to ranibizumab initially, complete fluid absorption was achieved after switching to 3 loading aflibercept injections (Fig. 1). Photodynamic therapy (PDT) was applied in the other 2 eyes and 4 eyes which did not respond to aflibercept initially, and dry macula was noted at 3 months after the PDTs in all 6 eyes. Recurrent fluid accumulation was not noted in these 6 eyes by the end of a 1-year follow-up period.

**Figure 2:**
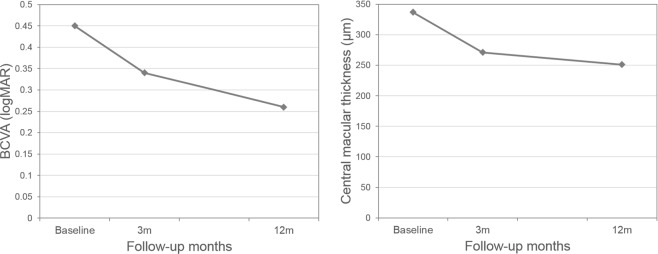
Changes in best-corrected visual acuity (BCVA) and central macular thickness (CMT) during the 12-month follow-up period. The mean BCVA and CMT improved at 3 months after three loading anti-vascular endothelial growth factor injections, and after the treatment switch, those outcomes further improved at 12 months.

Including the treatment switch, the total mean BCVA of 54 eyes gradually improved from 0.45 ± 0.25 logMAR at baseline to 0.34 ± 0.27 logMAR at 3 months and to 0.26 ± 0.25 logMAR at 12 months (P < 0.001, P < 0.001, respectively). The mean CMT showed similar improvement from baseline (337 ± 53 µm) to 3 months (271 ± 69 µm, P < 0.001) and 12 months (251 ± 68 µm, P < 0.001). Figure 2 shows the BCVA and CMT changes at a 12-month follow-up.

## Discussion

In the current study, we first demonstrated that aflibercept was more effective in inducing fluid absorption in pachychoroid neovasculopathy at least in the short term. Reduction in SFCT was significantly higher in the aflibercept group at 3 months. Although visual outcome did not differ significantly between the two groups after 3 loading injections, the aflibercept-treated eyes showed a higher incidence of dry macula at 3 months. Switching to aflibercept also resulted in complete fluid absorption in 86.7% of poor responders to ranibizumab. Because the switching was done immediately after 3 loading injections, we can assume that this may have resulted from the difference in drug efficacy rather than from the development of tachyphylaxis to ranibizumab^12^.

To date, no randomized trials have compared aflibercept with ranibizumab for nAMD, apart from the pivotal phase III randomized controlled trials of aflibercept^9^. The proportion with dry retina at 1 year was similar between the two drugs in the VIEW 1 trial; however, it was higher in the aflibercept group (80.3%) than in the ranibizumab group (60.4%) in the VIEW 2 trial in which one fourth of enrolled patients were not Caucasian. Also, a subgroup analysis of those trials showed that the proportion of dry macula at 3 months was greater for intravitreal aflibercept than for ranibizumab (75.7% versus 53.3%) in Asian patients^13^. These results may reflect that aflibercept can be superior to ranibizumab in eyes with thick choroid and CVH, which are prevalent characteristics in Asian populations^14^. In the current study, about two thirds of enrolled eyes demonstrated CVH on ICGA, which was observed in 44.4% to 53.8% of pachychoroid neovasculopathy in previous reports^4,5^. Several studies have reported that aflibercept was superior to ranibizumab in reducing choroidal thickness and achieving remission of exudation in eyes with thick choroid and/or CVH^15,16^. The reduction of SFCT was significantly greater in the aflibercept group, which may represent further reduction in CVH and disease activity.

The etiology of CNV development in pachychoroid neovasculopathy remains elusive and may be different from that in typical nAMD. Based on the observation of choriocapillaris attenuation at the disease’s focus in pachychoroid phenotypes^17,18^, it has been suggested that the neovascular process may be triggered by focal RPE disturbances and inner choroid attenuation overlying pachyvessels, which leads to up-regulation of VEGF. Others have proposed that chronic inflammation involving the choriocapillaris may also play a role in angiogenesis^19^. Hata *et al*. reported lower intraocular VEGF level in pachychoroid neovasculopathy than in nAMD^5^. Nevertheless, the authors reported that pachychoroid neovasculopathy responded favorably to anti-VEGF therapy, which was also demonstrated by the results of this study. In the current study, almost 90% of patients responded well to anti-VEGF drugs initially, and only a few patients (6 of 54 eyes, 11.1%) needed adjunctive PDT for complete fluid absorption. The favorable response to PDT in these eyes is in line with our previous report that showed the efficacy of adjunctive PDT in pachychoroid neovasculopathy refractory to anti-VEGF monotherapy^20^. In PCV, the primary treatment of choice is shifting from PDT to anti-VEGF injections due to the inherent risks associated with repeated PDT, including cumulative damage to normal choroidal vasculature and RPE^7^. To minimize the deteriorating effects, applying PDT as a deferred or rescue option is being attempted in PCV^21,22^, and the same strategy might be appropriate in pachychoroid neovasculopathy.

The concept of pachychoroid has evolved from the increase in choroidal thickness to the presence of characteristic morphologic changes (pachychoroid phenotype) that implicate structural and functional choroidal alteration. These include dilated choroidal vessels in Haller’s layer (pachyvessels), accompanied by thinning of the choriocapillaris and Sattler’s layer with or without RPE abnormalities overlying the pachyvessels. Eyes with pachychoroid disease may have normal SFCT if the luminal volume increase in the outer choroidal vessel is offset by a reduction in inner choroidal volume^3^. Indeed, our group previously reported that the SFCT of PCV exhibited a bimodal distribution, with peaks at 170 μm and 390 μm^18^. To date, however, there is no consensus on whether type 1 neovascularization in thin/normal choroid with dilated Haller vessels should be classified as pachychoroid neovasculopathy or nAMD in Asians. To investigate whether the two anti-VEGF agents may yield different treatment responses in pachychoroid neovasculopathy, we narrowed the inclusion criteria and minimized the gray zone. Thus, we only enrolled eyes with pachychoroid features and thick choroid (SFCT of 300 μm or more)^17^ in the current study.

The current study has several limitations, including its retrospective and nonrandomized nature. However, we collected a relatively large number of cases with strict inclusion criteria, and there was no significant difference in baseline characteristics between the two groups. All treatment decisions were made by a single retinal specialist (WKL) with a certain strategy. Because treatment switch was made after 3 loading injections, we cannot compare the efficacy of the two drugs in the long term. It is interesting that initial responders to both drugs showed favorable response and did not require treatment switch until 12 months later. Therefore, the treatment strategy of pachychoroid neovasculopathy needs to be individualized, and further studies should be focused on identifying possible prognostic factors predicting favorable treatment responses.

Despite the absence of clear consensus in defining pachychoroid neovasculopathy, we enrolled eyes exhibiting pachychoroid phenotypes with thick choroid to adhere to the original description of this specific disease. Based on our results, intravitreal aflibercept was superior to ranibizumab in reducing choroidal thickness and achieving dry macula at 3 months, and was also effective to initial poor responders to ranibizumab. The exudation from pachychoroid neovasculopathy was successfully managed with either of the two anti-VEGF drugs in most eyes over a 1-year treatment period. However, further research with longer follow-up periods and comparing treatment regimens are still needed.

## Methods

We conducted a retrospective review of the medical records of treatment-naïve patients with pachychoroid neovasculopathy treated at the Seoul St. Mary’s Hospital of the Catholic University of Korea between January 2014 and October 2016. This study was approved by the Institutional Review Board of the Seoul St. Mary’s Hospital College of Medicine, which waived the written informed consent because of the study’s retrospective design and was conducted in accordance with the tenets of the Declaration of Helsinki.

### Inclusion and exclusion criteria

**Figure 3:**
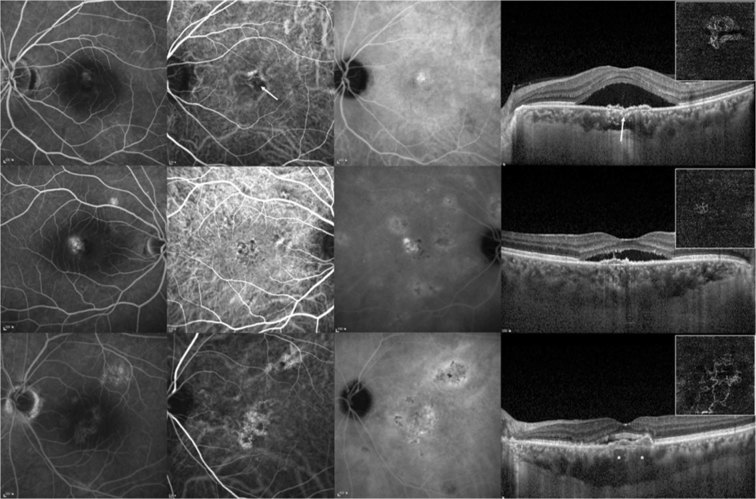
Multimodal imaging features of pachychoroid neovasculopathy. (Top row) Images of the eye of a 52-year-old female. Fluorescein angiography (FAG) shows minimal leakage, and indocyanine green angiography (ICGA) images show a small neovascular network on the early phase (arrow) and choroidal vascular hyperpermeability (CVH) in the mid-to-late phase. Spectral-domain optical coherence tomography (SD OCT) through the lesion demonstrates Type 1 neovascularization, which is apparent on swept-source OCT angiography (SS OCTA, inlet). The arrow indicates the presume site of feeder vessel ingrowth. The subfoveal choroidal thickness is 311 µm. (Middle row) Images of the eye of a 65-year-old male. FAG shows leakage at the fovea, while neovascular network is not evident on early-phase ICGA. Late-phase ICGA shows CVH at the posterior pole. A small pigment epithelial detachment is noted on SD OCT, which is revealed as choroidal neovascularization on SS OCTA (inlet). The subfoveal choroidal thickness was 520 µm while dilated Haller vessel is not apparent. (Bottom row) Images of the eye of a 68-year-old male. Late-phase FAG reveals ill-defined leakage. Abnormal choroidal vessels without polypoidal lesions are noted in the early-phase ICGA and CVH in the mid-to-late phase. SD OCT shows Type 1 neovascularization and the is 509 µm. Dilated Haller vessels (asterisks) are noted with loss of overlying choriocapillaris and Sattler layers below the area in and around the Type 1 neovascularization lesion. Choroidal neovascular network is evident on SS OCTA (inlet).

The diagnosis of pachychoroid neovasculopathy was based on the presence of Type 1 neovascularization in patients older than 50 years old without any features of AMD or other degenerative changes. The presence of Type 1 neovascularization was confirmed on swept-source OCT angiography (SS OCTA) at least once from baseline throughout the 12 months of follow-up. We enrolled cases that exhibited localized areas of choroidal thickening directly below the neovascular tissue and/or pachyvessels with attenuation of overlying choriocapillaris. Minimum cutoff value of SFCT was 300 µm. The enrolled patients were followed for at least 12 months after initial treatment. The exclusion criteria were presence of polyps on ICGA, drusen greater than Category 1 criteria used in the Age-Related Eye Disease Study^23^, presence of RPE tears or subretinal fibrosis, and the presence of any other ocular disease that may have affected visual acuity. Patients with serosanguinous changes associated with large pigment epithelial detachment, which is a frequent presenting feature of PCV even in cases without definitive polyps, were also excluded^24^. The representative cases are shown in Fig. 3.

### Treatment and assessment schedule

All patients initially received 3 monthly intravitreal anti-VEGF injections, using 0.5 mg of ranibizumab (Lucentis®; Genentech, Inc., South San Francisco, CA, USA) or 2 mg of aflibercept (Eylea; Bayer HealthCare, Berlin, Germany). Each patient was scheduled for a follow-up examination at 3 months, and complete fluid absorption was assessed at that time. Patients who showed complete fluid absorption at 3 months (initial responder) were followed regularly at 1- to 2-month intervals, depending on lesion activity. Retreatment was administered with initial anti-VEGF drugs in these patients whenever evidence of recurrent/ persistent fluid involving the fovea was detected on follow-up OCT (as needed). In patients with persistent intraretinal or subretinal fluid at 3 months (initial non-responder), the treatment switch was made at that time point. In patients who received ranibizumab, the treatment was changed to 3 monthly intravitreal aflibercept injections. In patients who received aflibercept initially, full-dose PDT was administered adjunctively. For PDT, patients were administered 6 mg of verteporfin per square meter of body surface area intravenously over 10 minutes. Fifteen minutes after the start of the infusion, PDT at standard fluence (light dose, 50 J/cm^2^; dose rate, 600 mW/cm^2^; wavelength, 689 nm) was applied for 83 seconds. The laser spot size was determined by adding 1 mm to the margin of the lesion detected on ICGA. Thereafter, all initial non-responders were followed up at 1- to 2-month intervals based on disease activity, and retreatment was given with aflibercept.

### Outcome measurement

Patient charts were reviewed until 12 months after the initial treatment. BCVA was measured using a standardized Snellen chart, and comprehensive ocular examination and SD OCT imaging with enhanced depth-imaging (EDI) mode was performed using Spectralis OCT (Heidelberg Engineering, Heidelberg, Germany) for both eyes at baseline and for the affected eye at each follow-up visit. A 25-line horizontal raster scan covering 30° × 20° centering at the fovea was obtained for each eye, and an individual B-scan was 240 µm apart from the next B-scan. CMT was designated as the average thickness of the 1-mm central area of the SD OCT map. SFCT was measured using EDI OCT at baseline and 3 months. We used the entire length of an EDI OCT scan for image binarization and measured the CVI by using the protocol described in previous studies^25,26^. The average of the two independent graders’ (JHL, JYK) measurements was used for analysis. The intra-class correlation coefficients for inter-observer variability about CVI were used to assess the reliability of the agreements. FAG, ICGA, and FAF (Heidelberg Retina Angiograph; Heidelberg Engineering) were performed at baseline in all patients. For the FAF image, a 30° × 30° area centered at the fovea was scanned using excitation with a 488-nm argon laser and 500-nm filter. CVH was defined as multifocal areas of hyperfluorescence with blurred margins within the choroid evaluated in the late phase of ICGA. Two retinal specialists (JHL, BJJ), who were blinded to all the other medical information, evaluated CVH and, in cases of discrepancy, another retinal specialist (WKL) made the final decision. For the inter-observer agreement evaluation about CVH, Fleiss’ kappa test was used. SS OCTA images were acquired using the DRI OCT Triton machine version 10.11 (Topcon Corporation, Tokyo, Japan). Acquisition protocol in the macular region consisted of a 3 × 3 mm^2^ area centered on the fovea for maximum resolution of the lesion examined. Whenever the lesion extended beyond the image border, a 4.5 × 4.5 mm^2^ or 6 × 6 mm^2^ area was used to include the entire extent of the lesion.

The main outcomes measured included the rate of complete fluid absorption at 3 months between the two groups and comparisons of visual and tomographic outcomes between baseline and 12 months in all enrolled cases. The recurrence rate and number of retreatments until 12 months was also evaluated within initial responders.

### Statistical analysis

For statistical analysis, Snellen visual acuity was converted to logMAR. The Mann-Whitney test was used for analysis of continuous variables, and the chi-square test was used for categorical variables. For comparison of BCVA and CMT at 3 months and 12 months with baseline values, the paired t-test with Bonferroni correction was used. Statistical analyses were performed using SPSS software (version 21.0; IBM Corp., Armonk, NY, USA). A P value of <0.05 was considered to indicate statistical significance.

## Data Availability

All authors agree to make materials, data and associated protocols promptly available to readers.
